# Effectiveness of Online Vaccine Communication Training and Assessment Using Virtual Encounters With Standardized Vaccine-Hesitant Parents

**DOI:** 10.7759/cureus.107736

**Published:** 2026-04-26

**Authors:** Shanna M Barton, Aaron W Calhoun, Carrie A Bohnert, Victoria A Statler, Nicole M Bichir, Michael L Bricken, Jennifer H Tasman, Sara M Multerer, Gary S Marshall

**Affiliations:** 1 Department of Pediatrics, Pediatric Infectious Diseases, Norton Children's Hospital, Louisville, USA; 2 Department of Pediatrics, Pediatric Infectious Diseases, University of Louisville School of Medicine, Louisville, USA; 3 Department of Pediatrics, Critical Care Medicine, Norton Children's Hospital, Louisville, USA; 4 Department of Pediatrics, Critical Care Medicine, University of Louisville School of Medicine, Louisville, USA; 5 Standardized Patient Program, University of Louisville School of Medicine, Louisville, USA; 6 Department of Pediatrics, General Pediatrics, Cincinnati Children's Hospital Medical Center, Cincinnati, USA; 7 Department of Pediatrics, General Pediatrics Gratis Faculty, University of Louisville School of Medicine, Louisville, USA; 8 Department of Pediatrics, Pediatric Hospital Medicine, Norton Children's Hospital, Louisville, USA; 9 Department of Pediatrics, Pediatric Hospital Medicine, University of Louisville School of Medicine, Louisville, USA

**Keywords:** online training, standardized patients, structured communication, vaccine hesitancy, virtual encounters

## Abstract

Background: There are no accepted best practices for counseling vaccine-hesitant parents, and targeted training in this area is not required during residency, despite widespread vaccine hesitancy among parents. To this end, much interest has focused on structured approaches to interacting with vaccine-hesitant parents. The Announce-Inquire-Mirror-Secure (AIMS) Method for Healthy Conversations is one such structured communication strategy that attempts to build trust between provider and parent, opening the door to receptivity to vaccination.

Objective: Determine if online training in a structured vaccine hesitancy communication strategy results in specific behaviors and increased confidence during telemedicine encounters with standardized patients (SPs).

Methods: Pediatrics and medicine-pediatric residents were randomized to receive either AIMS or control training online. Residents underwent pre- and post-training telemedicine encounters with SPs simulating a well-child immunization visit. Encounters were video-recorded and assessed using the Vaccine Hesitancy Communication Assessment (VHCA). Resident confidence and SP evaluations of the encounter were assessed pre- and post-training. Investigators, residents, SPs, and video raters were blinded to treatment allocation.

Results: From December 2020 to June 2021, a total of 54 of 58 (93%) eligible residents were enrolled in the study; 51 (94%) completed the study per-protocol, and 47 (92%) of these had evaluable video files. Resident self-confidence improved in both groups. AIMS behaviors were more commonly detected in AIMS than in control-trained residents. SP assessment of resident communication skills did not differ between groups.

Conclusions: Vaccine hesitancy communication training and assessment using SPs can be successful when implemented entirely in a virtual environment. Confidence gains were seen in both the AIMS and control arms, suggesting that this outcome resulted from the SP encounter itself. Similar online training and simulation programs could prove valuable as residents enter practice and the use of telemedicine expands.

## Introduction

Vaccine hesitancy, a state of mind regarding immunization marked by uncertainty, indecision, conflict, or opposition [1], is widespread and commonly encountered in everyday practice [2-4]. Historically, residency programs have struggled to prepare trainees for this reality [5-7]. Recognizing this, the Accreditation Council for Graduate Medical Education (ACGME) recently revised program requirements to ensure that residents learn effective communication strategies with those who are vaccine-hesitant [8,9]. However, the details on how to accomplish this goal were left to the individual program [7,10].

While there is no accepted best practice for communicating with vaccine-hesitant parents, the evidence is strongest for techniques that incorporate a presumptive approach, employ motivational interviewing, establish trust in the provider, and build collaborative parent-patient partnerships [11-15]. Much interest has focused on developing structured approaches to interact with vaccine-hesitant parents, with the advantage of being easy to remember and execute [16]. Our interest has been in a novel communication strategy called the Announce-Inquire-Mirror-Secure (AIMS) Method for Healthy Conversations [17-19]. Key elements include a presumptive, assertive opening statement that assumes a willingness to vaccinate (Announce); thoughtful inquiry to assess concerns (Inquire); verification of understanding using reflective statements (Mirror); provision of facts where appropriate (Respond, not included in the acronym); and affirming the relationship (Secure) [17-19].

Just prior to the COVID-19 pandemic, our group was conducting a blinded, pseudo-randomized, controlled study to determine if pediatric residents could be trained to employ AIMS during live encounters with standardized patients (SPs) portraying vaccine-hesitant parents [20]. Residents completed in-person pre- and post-training clinical encounters with blinded SPs; encounters were video-recorded and assessed by blinded raters using the Vaccine Hesitancy Communication Assessment (VHCA), a tool developed for the study. The study showed that behaviors of interest were more readily detected among AIMS than control-trained residents. The VHCA demonstrated low to moderate internal consistency and moderate inter-rater reliability. Self-reported resident confidence increased in both groups. SPs perceived no differences in aspects of resident performance such as respect, empathy, and promotion of trust. The study demonstrated that pediatric residents could be trained in AIMS and that an SP model of vaccine hesitancy could be used to assess learned skills.

During the early years of the COVID-19 pandemic, much of medical education shifted from in-person experiences to virtual learning [21,22], and some of the changes that occurred are likely to be permanent [23]. Virtual learning has many advantages, including asynchronous self-directed learning opportunities; cost-effectiveness; interactive technical features, including telemedicine; and ease of collaboration with outside institutions [21,22]. Prior studies have demonstrated that in a wide variety of contexts and content areas, from history-taking skills [24], physical exams [25], and procedures [26] to counseling [27] and communication skills [28-30], virtual training programs perform similarly to in-person models. Learner assessment can also be done in the virtual environment [21,31], and SPs can be a part of the virtual learning world [24,25,30,32,33].

The social restrictions imposed during the COVID-19 pandemic necessitated that our study be transitioned entirely to a virtual environment. The primary objective of this study was to determine if the transition to virtual AIMS training and telemedicine encounters with SPs would result in outcomes similar to those seen with live training and encounters, namely specific behaviors during telemedicine encounters with SPs and gains in confidence. Secondary objectives were to improve the performance of the VHCA and to modify the SP portrayal to more faithfully represent the well-meaning parent who is reluctant to vaccinate but is willing to listen.

This article was previously presented as a poster presentation at the 60th Annual Meeting of the Infectious Diseases Society of America in Washington, DC, on October 20, 2022. This article was previously posted to the Research Square preprint server on March 11, 2025.

## Materials and methods

SP model of vaccine hesitancy

For the current study, the SP case materials were modified, shifting the SPs’ posture from vaccine refusal to well-intentioned concern and reluctance to vaccinate; the goal was to more closely approximate what is encountered in real-world practice (Appendix 1). SPs were told to assume beliefs and thoughts that align with parents who might eventually accept vaccination. In the modified scenario, the infant had received vaccines at birth and at two months, but since then, the parent had heard and read conflicting information, prompting them to question the four-month vaccinations. At the conclusion of the encounter, SPs could decide whether or not to accept vaccination based on their interactions with individual residents. Pre- and post-training encounters utilized different SPs so as not to confound outcomes based on familiarity. 

SPs who participated in the initial study were not employed for the current study. Candidate SPs were interviewed by one member of the study team, and those with pre-existing bias against vaccines were excluded. In the end, six SPs were chosen, and all were trained on the case materials in a one-hour in-person training session conducted by two members of the research team. The in-person training session included a review of the case materials; a discussion of communication and behavioral parameters, including the range of affective presentation deemed appropriate for the role; and a practice session wherein the research team members acted out the role of the SP and resident. All SPs then viewed an in-person encounter from the pilot study. Independent study of the case materials was encouraged, such that each SP could individualize role portrayal. Following training, SPs completed the modified eight-item Vaccine Hesitancy Scale (VHS) [2], a validated parent survey, in order to screen for and exclude outliers (none were detected). Throughout the study, four SPs assigned to the project rotated between portraying the vaccine-hesitant parent and watching fellow SPs in simulation to ensure adherence to role portrayal. Standardization of role portrayal was assessed using the Maastricht Assessment of Simulated Patient (MASP) [34]. Two of the SPs served as moderators and proctors but did not engage in clinical encounters; these individuals provided additional quality assurance regarding role portrayal. SPs were blinded to participants’ year of residency training, group allocation (AIMS or control), and whether the encounter was pre- or post-training.

SPs completed an online survey immediately after the encounter, assessing their perception of the resident’s communication skills (Appendix 2). This questionnaire was revised from its original version in the pilot study to include open-ended questions. SP case materials and the SP online survey were developed by investigators for use in this study; therefore, specific licensure was not indicated.

VHCA

The VHCA underwent iterative review by three investigators involved in content development based upon input from the raters who participated in the initial study. Psychometrics were calculated on the VHCA, which indicated low to moderate internal consistency (Cronbach’s alpha of 0.31 to 0.65 performed per rater) and moderate inter-rater reliability (intraclass correlation of 0.63, two-way random effects, average rater, and absolute value). Given the need for higher inter-rater reliability if the VHCA is to be reliably used for research purposes, factor analysis, subsection-level inter-rater reliability, and item-level agreement calculations were performed to determine whether the removal of specific items might further improve reliability. Factor analysis revealed a two-factor solution, but subsection-specific mapping of these factors varied substantially between raters. Likewise, subsection-specific inter-rater reliability varied between 0.45 (phase 5) and 0.71 (phase 1), and item-specific correlations revealed substantial differences in rater scoring on an item-to-item basis, with correlations ranging from 10% (phase 3 item 4) to 95% (phase 2 item 5). A pattern, however, was difficult to discern from this data. For that reason, we adopted a more wholesale approach to VHCA revision in which each item was considered for exclusion or revision depending on item-specific correlation scores (a threshold of 60% or less was chosen for initial consideration), the opinion of the raters from the first phase on the importance and ease of interpretation of the item, and the opinions of the study authors as to the observability of the behavior in question and its centrality to the AIMS model. The revised VHCA (Appendix 3) consisted of five subsections, each corresponding to the “phases” of the AIMS model. The revised VHCA then underwent formal testing by new blinded raters who differed from those in the initial study. The VHCA was developed by investigators for use in this study; therefore, specific licensure was not indicated.

Participants, group assignment, and blinding

Participants included pediatric residents and internal medicine-pediatric residents at the University of Louisville School of Medicine. The curriculum consisted of one online module detailing the basics of vaccinology (Appendix 4); one online module reviewing communication strategies with vaccine-hesitant families (AIMS or control; Appendices 5 and 6, respectively); video-recorded SP encounters before and after training; and a final feedback session. Feedback was provided by two blinded general pediatrics faculty members not involved in study design and was not specific to AIMS-related behaviors. Completion of the curriculum was required by the pediatric residency program. Consent to participate in the investigational aspect of the study additionally included completion of two brief surveys and permission to have the video-recorded SP encounters confidentially reviewed and scored by blinded study personnel. Residents were randomized to either AIMS or control training and blinded to their group assignment. A summary table detailing the changes from the in-person pilot study to the virtual study is provided in Appendix 7.

Procedures

**Figure 1:**
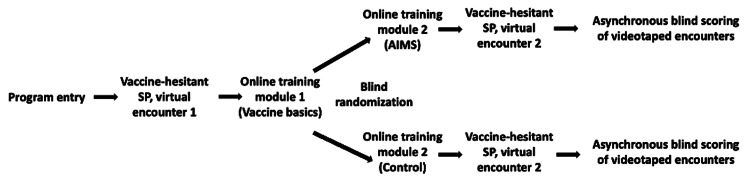
Study procedure Flow chart depicting the study procedure for residents in the AIMS- and control-trained arms. SP: standardized patient; AIMS: Announce-Inquire-Mirror-Secure

This study was conducted as a prospective randomized interventional trial. Study design is pictured in Figure 1. All encounters were video-recorded, analyzed, and independently scored by three raters using the VHCA. Each rater was a general pediatrician who had not participated in the initial study and completed virtual training on the VHCA prior to video scoring; faculty who served as raters differed from those who provided resident feedback. Save for the one investigator who was responsible for randomization, all remaining investigators, residents, SPs, and video raters remained blinded to group allocation.

Outcome measures

The principal outcome measure was the detection of AIMS-related communication behaviors in the AIMS and control groups. A secondary outcome was resident self-assessment of confidence in both groups. In addition, the revised VHCA was assessed for reliability, and SPs’ trust in the providers was measured.

Statistical analyses

Preliminary calculation demonstrated a minimum of 20 residents per group would be needed to detect a 10% or greater absolute change in VHCA total score with a power of 0.8 and an alpha value of 0.05. This calculation was performed under the assumption that the VHCA is similar to other typical assessment tools with comparable standard deviations in score.

VHCA psychometrics mirrored those utilized in the initial study. Cronbach's coefficient alpha was calculated for each rater to assess internal consistency. The two-way random effect model, absolute agreement, and average rater intraclass correlations were calculated for the VHCA as a whole to assess inter-rater reliability.

Pre-training and post-training VHCA and the SP encounter survey were analyzed using the Mann-Whitney U test. Pre- and post-training resident confidence survey scores were analyzed using analysis of covariance (ANCOVA) with rank transformation to ensure normal distribution of data. The effect size for each of these comparisons was calculated using partial eta squared.

All analyses were performed by a member of the research group who was blinded to participant group allocation. All calculations were performed using the IBM SPSS Statistics for Windows, Version 28 (Released 2021; IBM Corp., Armonk, New York, United States).

Ethics approval and consent to participate

This study was conducted in accordance with the principles outlined in the Declaration of Helsinki. The study was approved by the University of Louisville Institutional Review Board, whereby completion of the initial survey requesting resident demographic information and opinions about the existing vaccine education curriculum served as an unsigned informed consent document (IRB number: 20.0885; reference number: 717391; approved 11/18/2020). Informed consent was obtained from all study participants.

## Results

From December 2020 to June 2021, a total of 54 of 58 (93%) eligible residents were enrolled in the study; 51 (94%) completed the study per protocol, and 47 (92%) of these had evaluable video files. Baseline demographics of AIMS and control-trained residents were similar, including their assessments of the vaccine education they had received during residency (Table 1).

**Table 1:** Baseline characteristics PGY: postgraduate year; AIMS: Announce-Inquire-Mirror-Secure *values are median (interquartile range); **type of vaccine education received during residency (1 = strongly disagree; 5 = strongly agree)

	AIMS		Control	
Characteristic	Value	N	Value	N
Age	28 (27,30)*	26	28 (27,29)	25
Pediatrics		18		20
Internal Medicine-Pediatric		8		5
Year of graduation	2020 (2019,2020)*	26	2019 (2018,2020)	25
PGY1		16		12
PGY2		6		7
PGY3		3		6
PGY4		1		0
Received informal vaccine education during residency**	4 (4,4)**	26	4(4,4)	25
Received formal vaccine education during residency	3 (2,3.75)	26	3 (3,4)	25
Received vaccine hesitancy education during residency	3 (3,4)	26	4 (3,4)	25

**Figure 2:**
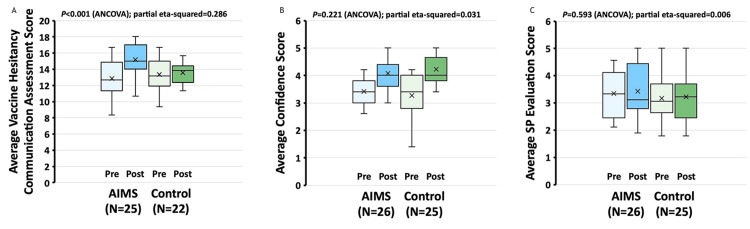
Detection of AIMS behaviors, resident self-confidence, and SP post-encounter survey Box and whisker plots showing pre- and post-training Vaccine Hesitancy Communication Assessment Score (which assesses AIMS behaviors) (A), confidence score (B), and SP evaluations (C). Upper and lower quartiles are marked by the top and bottom of the boxes. The median is marked by a horizontal line inside the box, and the mean is shown by the “x.” Whiskers indicate upper and lower limits. AIMS: Announce-Inquire-Mirror-Secure; SP: standardized patient; ANCOVA: analysis of covariance

After controlling for pre-training skill levels via ANCOVA, a statistically significant increase in median total post-training VHCA score was detected in the AIMS group (pre 12.7 (IQR 11.3-14.8), post 15.0 (14.0-17.0)) but not in the control group (pre 13.2 (11.9-15.0), post 13.8 (12.3-14.4)) (P < 0.001; Figure 2A); the effect size was moderate (partial eta-squared 0.286). The median confidence score increased in both groups (AIMS pre 3.4 (3.0-3.9), post 4.0 (3.6-4.4); control pre 3.4 (2.8-4.0), post 4.0 (3.8-4.7)) (P = 0.221, partial eta-squared 0.031; Figure 2B). SP assessment of the participants’ communication skills did not differ between AIMS and control-trained residents and did not vary from pre- to post-training (P = 0.593, partial eta-squared 0.006; Figure 2C).

The revised VHCA demonstrated internal consistency (Cronbach alpha) ranging from 0.32 to 0.59, and overall inter-rater reliability measured using intraclass correlation was moderate with similar pre- (0.52) and post-training (0.51) scores.

## Discussion

Ours is not the first study to utilize the virtual environment to deliver meaningful education on communicating with vaccine-hesitant patients or parents. Methods beyond the standard didactic module have included real-time question-and-answer sessions [29], role-play videos modeling effective communication [29,35], gamification [29], immersive virtual reality [36,37], supplemental reading with a guided questionnaire [29], or a smartphone application advising on recommended behaviors [37]. We are aware of only one other study that utilized virtual vaccine-hesitant SPs as a component of online training or assessment [38]; this study did not directly incorporate a structured approach to vaccine hesitancy communication, nor did it factor in participants’ baseline communication skills as a contributing factor to the acquisition of learned skills or engendering SP trust. Moreover, that study was limited to COVID-19 vaccine hesitancy. 

The current study realizes the full potential of the virtual environment by combining online training using a structured communication strategy and virtual patient encounters. Our results were similar to those in the initial study, making the case that training and assessment goals in this area can be achieved virtually. It is important to note, however, that sample size and study design do not permit direct quantitative assessment of non-inferiority between the two environments. Nevertheless, the overall equivalence of these results suggests similar functionality.

A formal, comprehensive virtual curriculum in vaccine hesitancy communication like the one described here has many advantages. Residents may benefit from the opportunity to practice their skills with SPs in a low-stakes environment using a format that mirrors contemporary telemedicine services [39]. To underscore the latter point, it is noted that telehealth competency has been added as an Entrustable Professional Activity (EPA) for pediatric residents and subspecialty fellows [23] and is supported by the American Medical Association (AMA) [40]. Moreover, the use of objective measures of skill acquisition may meet requirements for educational outcome metrics. Finally, a virtual curriculum like this one opens up the possibility of collaboration with other programs, including those that may not have access to an SP lab.

After completion of this study, we adopted the AIMS arm of the protocol as a standard element of our residency training program. While we acknowledge that there are no empirical data yet showing that use of AIMS improves immunization rates in the real world, we see the training to be as good as standard education, with the added benefit of uniform education regardless of clinic environment, patient population, and preceptor. In addition, a structured communication strategy may be of benefit to some residents who seek an accessible mnemonic and roadmap when involved in difficult patient encounters. Since confidence improved in both groups, it is possible that any specific training in vaccine communication will boost confidence. Moreover, since residents in both groups underwent encounters with SPs, it is also possible that the practice afforded by those encounters themselves, independent of training, elevated resident confidence.

This study has several strengths. First, it represents a unique deployment of hesitancy-displaying SPs to study vaccine-related communication using online encounters. Second, subjects were randomly assigned to treatment or control. Third, subjects, SPs, and raters were blinded to the intervention.

This study had several limitations. First, despite modifications to the VHCA, inter-rater reliability and internal consistency were largely unchanged compared to the initial study (note that because of these modifications, the VHCA scores in this study cannot be directly compared to those in the initial study). The continued low internal consistency is not unexpected given the heterogeneity of AIMS elements. In addition, many of the behaviors included in the AIMS model can be performed in a number of different, but no less effective, manners, potentially impacting how raters perceive them and thus inter-rater reliability. It is also possible that the low Cronbach's alpha stems from artifacts within the structure of the tool itself, which, if so, could render the statistical significance of our main findings less meaningful. However, the process by which the VHCA’s content was developed makes this less likely. Second, despite softening the SP portrayal, SPs were not able to differentiate AIMS-trained from control-trained residents. This indicates that AIMS behaviors, which we know were present as detected by the VHCA in blinded video reviews, were not perceived by SPs to be different from other communication behaviors. One possible explanation is that AIMS behaviors are not, in fact, different from standard of care behaviors in so far as engendering trust is concerned. Another possible explanation is that SPs as trust barometers are not sensitive or organic enough, in the context of these online, isolated, prescribed, vaccine-hesitancy encounters, to react genuinely to the nuanced differences between AIMS and control training. In this light, what matters is how vaccine-hesitant parents in the real world might react to AIMS communication, something that this study was not designed to address. Third, generalizability to other residency programs is limited because this was a single-institution study with a small sample size. Fourth, the results should be interpreted with caution, given the psychometric limitations of the VHCA. Finally, whereas this study suggests that the wholly online program was as effective as the in-person program, there were no predefined criteria for non-inferiority, and statistical comparisons are not possible because some study elements (e.g., VHCA and SP portrayal) were altered in the online program.

## Conclusions

Despite the medical achievements of vaccination, vaccine hesitancy has become increasingly common in clinical practice. As a result, residency programs have been tasked with ensuring graduating residents are adept at managing complex conversations with vaccine-hesitant families. However, details of the curriculum remain up to the discretion of an individual program and available resources, which is likely to result in variable resident training experiences. Virtual learning, trainee assessment, and telemedicine encounters have become increasingly commonplace in medical education following the COVID-19 pandemic and have previously been utilized as a component of vaccine hesitancy communication training. However, to our knowledge, ours is the first study to demonstrate that online vaccine hesitancy communication training using virtual encounters with SPs portraying vaccine-hesitant parents can effectively be used to assess resident performance in a manner similar to in-person environments and provide practice that might contribute to improved resident confidence. This approach also has the potential to improve training in the use of telemedicine and provide flexibility for residency programs in more geographically remote locations or that do not have ready access to simulation centers.

## Appendices

Appendix 1

Modified Standardized Vaccine-Hesitant Parent Case Materials

The purpose of this standardized patient (SP) encounter is to provide resident physicians with the opportunity to interact with, interview, and counsel a parent whose child is due for immunizations. You, the SP, are asked to play the role of a parent who presents to the clinic for their child’s routine well-child check. An infant mannequin in a car seat is provided for the encounter in order to simulate the presence of a child. Participating residents will be in all stages of residency training, from internship to senior resident. You will not know the resident’s level of training unless the resident chooses to disclose this information. Each resident will have an encounter with two different SPs over the course of a few weeks. If you are assigned a resident with whom you have already had an encounter, immediately stop the encounter and alert the proctor. Each encounter will take place within the allotted 15-minute time span.

The encounters will take place on a virtual platform simulating an office exam room; every effort should be made to replicate a live interaction in a physical space. SPs will be given a virtual background to use during the encounter to simulate an exam room. Residents will be provided with a “door chart” with pertinent patient information prior to entering the “room” (quotations here indicate the virtual nature of the items). The infant mannequin’s presence should be visible and known to the resident during the virtual encounter. While the scenario is that of a comprehensive four-month-old well-child check, residents are instructed to focus the discussion on the child’s immunizations. The primary goals of this encounter are for the resident to discuss the importance of vaccines to your child’s health, address concerns, and encourage you to vaccinate your child. 

As these clinical encounters are part of a research study, we ask that SPs not share their thoughts, experiences, impressions, or specific scenarios with anyone else, including other SPs.

Background information: You are playing the role of an average, well-meaning, fully engaged parent who has some concerns about vaccination. Your spouse is unable to accompany you to the office visit due to work obligations. You are the parent of one child, John Smith, who is four months old. John is an established patient in the clinic and is returning today for a previously scheduled routine four-month-old well-child check. Overall, John is in good health and in every respect is a normal baby; you do not have any specific questions or concerns regarding his nutrition, growth, development, or sleeping habits. John received the routine hepatitis B vaccine at birth and all of the recommended vaccines at two months of age. Since that time, you have heard conflicting information about vaccines from one or two family members, and you have read a few concerning things on social media. You are well-educated and reasonable, and you definitely understand the personal (prevention of disease in your child) and societal (prevention of disease in the community) benefits of vaccination. In fact, you received the Tdap and flu shot during pregnancy in order to protect yourself and your baby. You have some understanding of science, and you are skeptical of the “anti-vaxxers” who oppose all immunizations.

At the same time, you cannot ignore the things you have heard and read, and this has made you a little apprehensive about the four-month shots. In your heart of hearts, you just want to make sure you are making the right decisions for John.

This encounter gives you the opportunity to ask questions, voice concerns, and make a more informed decision regarding vaccinating John. That decision, whether or not to allow John to have his four-month shots, is purposefully left unscripted to give you an opportunity to decide based upon the conversation with each individual resident.

Child’s medical history: Birth, term infant. The mother received routine prenatal care, and there were no pregnancy complications. Spontaneous vaginal delivery without complications. Past medical history: no past or current medical problems and no hospitalizations; medications: none; allergies: none; surgical history: circumcision; family history: no relevant family medical history. Social history: Born in Louisville, KY. Does not attend daycare. Immunization history: Received hepatitis B vaccine at birth and two-month vaccines (DTaP-HepB-IPV combination, PCV13, Hib, and rotavirus)

Thoughts to bring to the encounter: You believe that immunizations protect children from disease. Here are four points of which you are aware:

1. Before vaccination programs were instituted, diseases like measles and polio claimed or adversely altered the lives of tens of thousands of children in the U.S. Today, as a result of vaccines, these diseases are rare. You do not want your child to get these diseases. 

2. There is a rigorous, reliable system for evaluating vaccine safety and efficacy. The bar for safety is much higher than that for drugs, because vaccines are given to healthy people, and our tolerance for risk is very low. The process involves large, meticulously-conducted and scrutinized clinical trials, approval by the FDA, and recommendation by authoritative bodies like the CDC and American Academy of Pediatrics. Those bodies have the best interests of children and communities in mind, and you reject conspiracy theories that say otherwise.

3. Doctors and medical organizations strongly recommend that all children receive all of the vaccines in the prescribed schedule, which protect against 14 different childhood diseases.

4. Some children have medical conditions that prohibit them from being vaccinated; their only path to protection is through community immunity-that is, the children around them being immunized.

However, you do have some concerns: While no harm came to John after the two-month shots, it seems in retrospect like a lot to give to one small child. Are we sure a baby’s immune system can handle all of that at one time? To this point, you know that some of your friends are using an “alternative” schedule for their children, where the shots are spread out over a few weeks or months

You are well aware of the numerous studies showing that vaccines do not cause autism, and you have read that every major medical organization, from the CDC to the AAP and WHO, have said that there is no connection. Yet, you’ve heard a few compelling stories from parents of autistic children who describe abrupt changes in their previously normal children right after getting the shots. You are having trouble reconciling the scientific studies with the anecdotal stories.

At the suggestion of a close friend, you read the package insert for the pneumococcal vaccine (PCV13). In addition to the vaccine antigen itself, the ingredients include aluminum phosphate, polysorbate 80, and succinate buffer. You want to understand why these substances are in there and that they are safe for John to receive.

Your posture during the encounters: You should be willing to share your questions and concerns and be receptive to discussion with the resident. You should listen to what the resident says and be mindful of how it makes you feel. You should exhibit decisional conflict about vaccination, not opposition to vaccination nor blind acceptance of the recommendations. You should be open to moving towards vaccination if you feel comfortable, or away from vaccination if you do not. At the end of the encounter, it is OK for you to accept the four-month shots for John. It is also OK for you to defer vaccination, opt for an alternative schedule, or even refuse vaccination. This is entirely up to you and should be dependent on the relationship you form with the resident during the encounter. The encounter will be limited to no more than 15 minutes; be cognizant of this and let yourself move in one direction or another in that time frame.

Keep in mind that each resident you encounter will have a different approach to this issue. So, it is OK to accept vaccination with one resident and defer vaccination with another. Also, remember that residents are early in their medical careers and may not have as much knowledge as more experienced physicians. Try not to focus too much on what they know in terms of hard facts; rather, allow yourself to be swayed to some extent by their tone, style of communication, and connection with you.

After learning this script and posture, you are free to explore the issue using the Internet and other sources, as you might in real life, before visiting the pediatrician. However, this script is not that of a “militant anti-vaxxer”, or even a “die-in-the-wool skeptic”. It is, rather, a script representing the majority of parents who are intelligent and just want to make sure they do the right thing for their children.

SP’s should be aware that their decision regarding vaccination does not translate into a score for the resident, i.e., acceptance does not equate to a “passing score” and vice versa. Therefore, this should not be a consideration during the clinical encounter. SP’s should be conscious of the potential for bias by simply agreeing to immunization because they feel it is consistent with the aim of this study. SP’s should be mindful that while the outcome regarding the SP’s decision to vaccinate will be noted, it is not the only factor being evaluated in this study.

At the conclusion of the encounter, SP’s should log off of the virtual platform. SP’s should not break character at the end of the encounter. Feedback will not be provided to the residents.

Appendix 2 

*Post-encounter Standardized Patient Vaccine-Hesitant Parent Questionnaire**     *      

Date: ____________

Standardized Patient Name: ___________

Resident Name: __________

Gap Kalamazoo Communication Skills Assessment Form*

Parent/Family Assessment

Questions should be answered on a 1-5 scale as follows: 1 = poor, 2 = fair, 3 = good, 4 = very good, 5 = excellent

A list of example statements that exemplify the behavior is included for each item. However, each item should be viewed as a whole, and only a single score should be given.

How well did your family member's clinician do at...

1. Builds a Relationship:

- The doctor was really interested in my family.

- The doctor's words showed that he/she cared for my child.

- The doctor seemed to care about our feelings and what we wanted.

- The doctor's body language showed that he/she cared for my child.

2. Opens the Discussion:

- The doctor let us finish things we had to say without interrupting.

- The doctor asked us about other things that might be worrying us.

- The doctor clearly explained why we were meeting.

3. Gathers Information:

- The doctor didn't try to force the conversation with his/her questions.

- The doctor asked us for more details about things we said.

- The doctor would occasionally repeat back what we had said as a summary.

- The doctor did not seem to interrupt us as he/she asked their own questions.

4. Understands the Patient's and Family's Perspective:

- The doctor asked about parts of our lives and personal histories that would affect our health.

- The doctor showed interest in our personal beliefs and concerns.

- The doctor asked what we thought about the treatments and tests being done.

5. Shares Information:

- The doctor asked what we understood about our child's illness.

- We understood the words our doctor used to describe our child's illness.

- The doctor would check to see if we had any questions after each explanation.

- The doctor gave us enough time to think about what he/she had said before moving on.

6. Reaches Agreement:

- The doctor included us in all the decisions that were being made.

- The doctor made sure that we understood what the next step would involve.

- The doctor asked what our feelings were about those plans before making any decisions.

- The doctor brought in outside help when we needed it (Social Work, Pastor).

7. Provides Closure:

- The doctor made sure that we had no more questions before leaving.

- The doctor gave a summary at the end of what we had talked about.

- The doctor set a time to meet again.

- The doctor told us who to call if we had more questions.

- The doctor showed a real interest in our family as people as he/she ended the meeting.

8. Demonstrates Empathy:

- The doctor showed compassion for our family.

- The doctor seemed to understand how we were feeling.

- The doctor responded to how we felt in a way that made sense to us.

9. Communicates Accurate Information:

- The doctor clearly explained our child's condition.

- The doctor clearly explained what our options were.

- The explanations our doctor gave were good enough for us to make important decisions.

10. What did this doctor do best at? (Please pick 3 choices):

- Builds a Relationship

- Opens the Discussion

- Gathers Information

- Understands the Patient's and Family's Perspective

- Shares Information

- Reaches Agreement

- Provides Closure

- Demonstrates Empathy 

- Communicates Accurate Information

11. Why did you choose those particular answers?

12. What could this doctor improve on? (Please pick three choices)

- Builds a Relationship

- Opens the Discussion

- Gathers Information

- Understands the Patient's and Family's Perspective

- Shares Information

- Reaches Agreement

- Provides Closure

- Demonstrates Empathy 

- Communicates Accurate Information

13. What could they have done better?

*Adapted from [41]

SP Post-encounter Open-Ended Questions

The following questions are designed to allow you, the SP, to reflect upon the clinical encounter with the resident and thoughtfully provide feedback on your experience. There are no “right” or “wrong” answers. You are encouraged to include your personal feelings and opinions in your answers. Where possible, specific examples of the encounter should be included in your answer. 

1. In this clinical encounter, did you choose to accept vaccination, defer vaccination, refuse vaccination, or some alternative outcome? Why?

2. In your opinion, what aspects of the resident’s communication strategy did you find most helpful?

3. Acting as a vaccine-hesitant parent, what aspects of the conversation enabled you to establish trust in the resident?

4. Considering factors other than the resident’s knowledge base, what aspects of the encounter might the resident change in order to establish a more trusting and receptive relationship?

Appendix 3

Modified Vaccine Hesitancy Communication Assessment

This tool is designed to assess communication between providers and vaccine-hesitant parents during face-to-face encounters. Behaviors of interest are framed according to the “AIMS” model, which stands for Announce, Inquire, Mirror, Secure-distinct phases that are described below and in the corresponding reference manual. One additional phase, Respond, is included here; unlike the other communication phases, Respond is heavily dependent on the provider’s knowledge base. This being said, there are behaviors of interest in this phase that the tool is designed to capture. Each phase is assessed by a series of Yes/No questions (raters should ignore the numerical scoring (“Yes = 1”, “No = 1”) in each phase).

At various points during real-life encounters, a parent might choose to accept vaccination, and the scoring sheet is designed to capture this. 

**Table 2:** Modified vaccine Vaccine Hesitancy Communication Assessment form

Element 1: Announce - Clear articulation at the start of the encounter that vaccines are due and should be given	
Score (Yes = 1)	Positive Behaviors
Yes/No	Provider introduced topic of vaccination by stating that "shots" (or equivalent) are due today
Yes/No	Provider declared early-on his or her intent to vaccinate the child at this visit
Score (No = 1)	Negative Behaviors
Yes/No	Provider affirmatively gave option to defer some or all vaccines, or conceded to deferral, during the introductory conversation
Yes/No	Provider exhibited hesitancy or uncertainty in introducing the topic of vaccination
Total =	
Notes	
Element 2: Inquire - Use of targeted questions to explore concerns expressed by the parent	
Score (Yes = 1)	Positive Behaviors
Yes/No	Provider focused his or her questioning on the highest priority concerns voiced by the parents
Yes/No	Provider communicated in some way his or her desire to fully engage the parent regarding these concerns
Yes/No	Provider explicitly asked for the parent's sources of vaccine information
Score (No = 1)	Negative Behaviors
Yes/No	Provider interrupted parent mid-sentence on 2 or more occasions
Total =	
Notes	
Element 3: Mirror - Clear and concise reflection of concerns back to the parent	
Score (Yes = 1)	Positive Behaviors
Yes/No	Provider accurately reflected the content of each high-priority concern back to the parent in subsequent conversation
Yes/No	Provider clearly communicated that they heard the parent's concerns
Yes/No	Provider made sure that his or her understanding of the parent's concerns was correct (individually or grouped)
Score (No = 1)	Negative Behaviors
Yes/No	Provider appeared to validate at least one concern that is generally regarded by the scientific community as having no basis in fact
Total =	
Notes	
Element 4: Respond - Evidence offered in genuine fashion to share knowledge and mititgate concerns	
Score (Yes = 1)	Positive Behaviors
Yes/No	Provider offer at least one fact-based reassurance for the highest-priority concerns
Yes/No	Provider explictly directed the parent to valid, authoritative resources regarding his or her vaccine concerns
Yes/No	Provider explained the known, common side effects of vaccination
Score (No = 1)	Negative Behaviors
Yes/No	Provider deferred to authoritative recommendations as a way of dismissing greater or equal to 1 specific concern, without providing scientific evidence to mitigate the concern or admitting a knowledge gap
Total =	
Notes	
Element 5: Secure - Offer to remain engaged in two-way dialogue regardless of decision regarding vaccination	
Score (Yes = 1)	Positive Behaviors
Yes/No	Provider made explicit, unconditional offer to discuss vaccination again
Yes/No	Provider acknowledged parent's interest in doing the right thing for their child
Yes/No	Provider conveyed the desire to maintain a therapeutic relationship with the parent
Score (No = 1)	Negative Behaviors
Yes/No	At least one of the providers' statements could be interpreted as dismissive
Total =	
Notes	
Outcome - Parent's position regarding vaccination at the end of the encounter (check one)	
Yes/No	Parent accepted all vaccinations that were due at this visit
Yes/No	Parent accepted at least one, but not all, vaccinations that were due at this visit
Yes/No	Parent refused all vaccinations that were due at this visit
Yes/No	Other
Notes	

Appendix 4

Module 1: Vaccine Communication Training Course

** **Format: PowerPoint-based online presentation by one of the investigators (Marshall).

Content included:

Basic Vaccinology

·      Adaptive humoral and cellular immunity

·      Role of the innate immune system

·      Types of vaccines

·      Correlates of protection

·      Herd immunity

·      Goals and impact of vaccination programs

Vaccine Practice

·      Clinical vaccine development

·      How recommendations are made

·      Schedule and platforms

·      Contraindications and precautions

·      Safety net

Common Concerns

·      Necessity of vaccines

·      Excipients and contaminants

·      Immune overload (too many too soon)

·      Allergies and autoimmune diseases

·      Autism

·      Demyelinating neurological disorders

Appendix 5

Module 2: Experimental AIMS (Announce, Inquire, Mirror, Secure) Training 

Format: Online pre-recorded PowerPoint-based lecture with Drs. Barton and Marshall.

Content included:

Discussing Vaccine Hesitancy

·      Introduce the AIMS Method for Healthy Conversations.

·      Announce: Introduce the need for vaccination and assume vaccination will occur using a presumptive assertive statement.

·      Inquire: If met with hesitancy, use open-ended questions to facilitate dialogue and elicit information in a neutral manner. Try to understand what drives the caregiver’s concern, evaluate the source and strength of their concern, and gauge the level of hesitancy.

·      Mirror: Utilize reflective listening as a means of demonstrating and verifying understanding. Be mindful not to restate caregiver concerns verbatim but instead reframe key points. Regardless of the validity of the concern, acknowledge the caregiver’s right to question. You may need to move through the inquiry and mirror stages several times before moving forward with the conversation.

·      Respond: Only once you have established a supportive environment and correctly identified the concerns should you move forward with providing a response. Consider the following when responding to a concern:

o   Use facts sparingly

o   Avoid saying there are “no” risks to vaccinating, but be honest and distinguish clearly between local reactions, severe, and very rare events. Explain the relative risk and discuss the risk of not vaccinating

o   Do not answer unasked questions, as this may trigger new concerns

o   Limit repetition of misinformation (e.g., myths) 

o   Direct the caregiver to valid and authoritative resources

o   Honestly acknowledge what you do not know 

·      Secure: Regardless of the outcome (e.g., acceptance, hesitancy, refusal), your goal is to establish a secure and trusting relationship. You may do this by reaffirming your position as a trustworthy teammate, maintaining respect and professionalism, establishing an open-door policy, and encouraging a follow-up appointment to discuss these concerns further.

Appendix 6

Module 2 - Control AIMS (Advocating for Immunization More Strongly) Training 

Format: Online pre-recorded PowerPoint-based lecture with a blinded general pediatrics faculty member.

Content included:

Discussing Vaccine Hesitancy

·      State which immunizations the patient is due for, using a presumptive approach

·      When met with hesitancy, use an open-ended question to elicit more information to figure out what the key concern is

·      Roll with resistance, validating that parents are mostly concerned about the safety and well-being of their child

·      Move into the facts about the benefits of immunization, and why the benefits of that immunization outweigh any risks associated with it

·      Address any other concerns or questions in a similar manner

·      Pause and read the body language of the parent to assess if the discussion has made any impact

·      If the parent is still not wanting to immunize, end on a positive note by offering more information on the immunization, noting that this can be discussed again at the next visit, or (if more time-sensitive, like a flu shot) they can return for a shot-only visit if they decide they want it.

Appendix 7

Outline of Changes From the Phase I (Pilot) Study

**Table 3:** Outline of changes from the Phase I (pilot) study

Study Element	In-Person (pilot)	Virtual
General		
Dates	September 2019-March 2020	December 2020-June 2021
Design	Pseudorandomized, blinded, controlled	Randomized, blinded, controlled
Subjects	Pediatrics residents	Pediatrics and Medicine-Pediatric Residents
Pre-training assessment of SP attitudes	None	Qualitative interviews
Post-training assessment of SP attitudes	None	Validated 8-item Vaccine Hesitancy Scale
SP posture	Adamant vaccine refusal throughout encounter	Hesitancy about accepting vaccination but receptive to discussion. Decision to accept or defer vaccination individualized to the resident-SP encounter
Intervention	AIMS (1-hour PowerPoint-based lecture provided in-person during weekday work hours and delivered prior to the second SP encounter)	AIMS (1-hour pre-recordeded PowerPoint-based lecture delivered asynchronously prior to the second SP encounter
Resident Assessment	In-person encounter with vaccine-hesitant SP in a mock exam room with exam table and infant mannequin. The encounter was conducted under the premise of an unimmunized 4-month-old infant presenting for a routine well-child appointment	Telehealth encounter via a virtual platform with a virtual background to simulate an exam room. Infant mannequin visible during encounter. The encounter was conducted under the premise of a 4-month-old infant who had previously received 0 and 2-month vaccines presenting for a routine well-child appointment
Outcome measures		
Behavioral outcome	VHCA	Modified VHCA
SP perception of encounter	Post-encounter Standardized Patient Questionnaire	Modified Post-encounter Standardized Patient Questionnaire
